# Real-Time User Identification and Behavior Prediction Based on Foot-Pad Recognition

**DOI:** 10.3390/s19132899

**Published:** 2019-06-30

**Authors:** Kuk Ho Heo, Seol Young Jeong, Soon Ju Kang

**Affiliations:** School of Electronics Engineering, College of IT Engineering, Kyungpook National University, 80 Daehakro, Bukgu, Daegu 702-701, Korea

**Keywords:** IoT Smart Home, user and behavior recognition, real-time identification

## Abstract

In the IoT (Internet of things)-based smart home, the technology for recognizing individual users among family members is very important. Although research in areas such as image recognition, biometrics, and individual wireless devices is very active, these systems suffer from various problems such as the need to follow an intentional procedure or own a specific device. Furthermore, with a centralized server system for IoT service, it is difficult to guarantee real-time determinism with high accuracy. To overcome these problems, we suggest a method of recognizing users in real time from the foot pressure characteristics measured as a user steps on a footpad. The proposed model in this paper uses a preprocessing algorithm to determine and generalize the angle of foot pressure. Based on this generalized foot pressure angle, we extract nine features that can distinguish individual human beings, and employ these features in user-recognition algorithms. Performance evaluation of the model was conducted by combining two preprocessing algorithms used to generalize the angle with four user-recognition algorithms.

## 1. Introduction

With the increasing availability of IoT (Internet of things), many studies of smart home–based IoT systems are being undertaken [1,2,3,4,5]. In particular, smart-home technology for the elderly has the potential to improve the quality of life in an aging society. IoT smart-home service for specific users such as senior citizens and dementia patients [6,7] should be able to distinguish between seniors who need customized services and other family members who need only regular services. Therefore, it is necessary to accurately determine who is in need of a service in real time and to provide enhanced services accordingly.

Typically, most studies in user recognition cover image processing and biometric technology, using human characteristics such as fingerprints or irises [8,9,10]. However, installing cameras in users’ homes raises issues such as encroachment on personal privacy, and biometric technology requires intentional procedures on the part of the user: physical contact such as a sensor device touching a finger or eye contact using an iris recognition sensor may be affected by users’ health conditions as well as environmental factors [10,11]. Most importantly, real-time performance degrades in low-memory and low-power embedded systems when users are recognized by image processing and deep learning, which are high-complexity computational techniques. Some research has used specific wearable devices [12] to avert this disadvantage, but such devices cannot distinguish who owns the device and are not appropriate for people who are likely to lose these devices or who tend to resist wearing them.

In order to overcome the disadvantages of existing research, we propose a system that measures foot pressure in real time and thereby recognizes users using a footpad consisting of pressure sensors. The system recognizes the user simply by their stepping on the footpad, without requiring them to perform a specific action or use a specific device. Therefore, this system assume that at least one foot is raised on the footpad. To enable user recognition from any angle, total least square [13] and center of gravity algorithms are used to measure the angle of foot pressure based on data from 48 horizontal and 48 vertical pressure sensors. By using the properties of the rotation matrix, we generalize the angle in the front of the pad and then extract features such as width and height of the user’s foot based on generalized foot pressure.

The system can configure the smart home-based IoT system using Bluetooth, wireless PLC and m-bus communication methods with various appliances in the house [3,4]. Also we can combine this system with the existing smart home-based IoT platform to create a smart home-based IoT platform for a specific users [5]. Therefore, due to the scalability of this system, many smart home-based IoT systems will be developed and used.

In this paper, we use low-complexity computation techniques for real-time user awareness in embedded systems, as opposed to the high-power computation techniques which have been used in existing user perception applications; Fuzzy theory [14], Gaussian Naive Bayes (GNB) approach [15], *k*-nearest neighbors (K-NN) algorithm [16], and artificial neural networks (ANNs) [17]. We were used to compare their performance (accuracy and performance time), and an optimal combination of algorithms was then derived.

This paper is structured as follows: Section 2 describes relevant existing research, Section 3 offers an overview of the whole system, and Section 4 presents the preprocessing methods employed. Section 5 compares the results of algorithm performance evaluation, and finally Section 6 offers some conclusions.

## 2. Related Research

As research in home IoT intensifies, many studies are being done on user recognition. Although user recognition using image processing such as facial recognition and also voice recognition [7,18,19] has been extensively studied, problems exist that are likely to cause errors depending on the conditions, including camera angle, the shape of the user’s head, and the user’s physical condition. Many studies have addressed these concerns, including studies on proximity-based neighbor identification protocol (PNIP) [12,20], which detects a low frequency device and transmits an advertising signal with the user’s ID, and radio-frequency identification passive (RFID passive) [21], a system that uses specific device and radio frequency (RF) to identify the user. However, PNIP protocols are problematic due to the need to carry or wear a specific device; for instance, they cannot reliably be applied to senior citizens with dementia or to children who are unwilling to wear the device, when the user lost wearable device or wear someone else’s wearable device, and malfunction due to device battery discharge, etc. Also RFID passive protocols has problem that the user has to intentionally touch the device to the reader.

To compensate for the defects of existing protocols, research is underway that recognizes users using foot pressure, an aspect of biometrics. User recognition studies based on foot pressure include footprint-based personal recognition [22] as well as personal recognition methods using either Bayesian algorithms or sequential walking footprints via overlapped foot shape and center-of-pressure trajectory [23]. For this last approach, the former technique acquires the shape of the foot and compares the similarity of the images using Euclidean distance, while the latter achieves a low error rate by adding a hidden Markov model to the dynamic footprint. However, because of the need for large amounts of sampling data and high computing power, a centralized server structure is indispensable and so predictability is low. Consequently, this method cannot guarantee definitive identification [24]. Therefore, in this paper we propose a cognitive system based on user foot-pressure distribution, which guarantees definitive results, comparing performance time and accuracy using several algorithms in Raspberry Pi 3 which is a low-specification embedded system platform that is easy to control because it has a Linux kernel, and it is a scalable platform such as USB, WIFI, GPIO, so it was used to build a user identification system quickly.

## 3. Overview of the Proposed System

The proposed scenario is as follows. First, a footpad consisting of 48 horizontal and 48 vertical pressure sensors is installed in front of user’s room in the home, including the bathroom, as well as home appliances such as a refrigerator and a gas stove. Then, when the user steps on the footpad, the angle of foot pressure is measured to determine the direction in which the user is likely to move.

**Figure 1:**
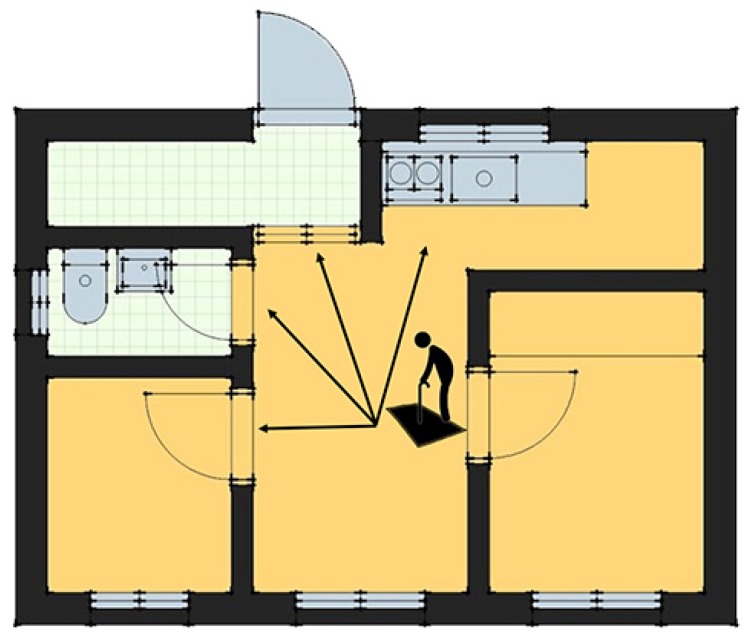
Suggested scenario for using a footpad in an IoT smart home.

For example, in Figure 1, if the user leaves the door and the angle of the user’s foot pressure distribution is between 270° and 300° (a clockwise angle that assumes the Raspberry Pi 3 and its joint is at 0°), the system decides that the user is going to go to the bathroom, whereas an angle between 0° and 30° is interpreted as meaning that the user is moving toward the kitchen. In addition, the system determines where the user is likely to go, simultaneously generalizing the pressure distribution, and then extracts particular features of foot pressure in order to recognize specific users and input feature values to the algorithm. Recognizing certain users in the family allows the IoT system to prevent children or elderly users with dementia patients from turning on gas fires, or to alert a guardian if these users have failed to flush the toilet. Furthermore, when calculating the time interval between footsteps as a user moves across a room, the system can inform the designated guardian when the user’s motion is slower than that user’s average pace. In this way, the home environment can be made safe for all family members.

**Figure 2:**
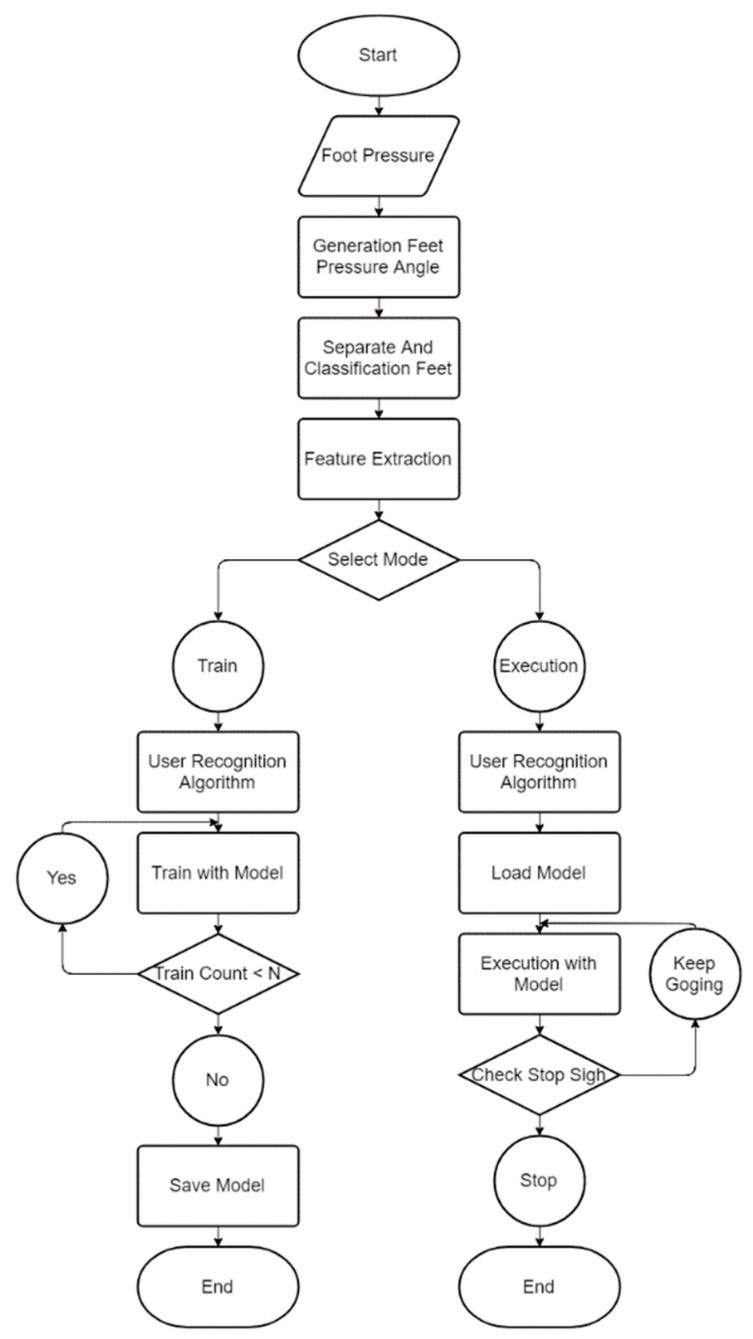
System flowchart.

Figure 2 shows a system flowchart schematizing how to determine the user’s identity and where they are likely to go next. The system consists of preprocessing, feature extraction, and learning and execution segments. The preprocessing part comprises two steps.

First, the angle of foot pressure is measured and generalized to obtain the consistent characteristics in foot-pressure feature extraction. Algorithm 1 shows the pseudocode representation for measurement and generalization of the pressure distribution angle. For this study, we use the foot-pressure center of gravity and total least squares algorithms for angle measurement. With the foot-pressure center of gravity algorithm, the centers of gravity at the front and heel of the foot are calculated using trigonometric functions. The results show a 180° discrepancy between choosing the criterion to be the center of gravity of the front part of the foot and the center of gravity of the back part, but in this study we were able to measure the angle of the foot correctly by locating the arch between the toe and sole of the foot and checking the foot’s orientation. The total least squares algorithm is derived from a linear function by minimizing the mean square distance between the foot pressure and the linear function, which may also result in a 180° error between calculating an angle clockwise versus counterclockwise from the criterion. The angle of the foot can be correctly measured by calculating clockwise from the arch of the foot. After measuring the angle, the difference was calculated based on the Raspberry Pi 3 and its joint, and the foot-pressure angle was then generalized using a rotation matrix [25].

**Algorithm 1.** Pseudo code for generalization of pressure distribution.
*Gravity_b_ = Center of gravity of ball of foot*

*Gravity_h_ = Center of gravity of heel of foot*

*Space_f_ = Space between toes and ball of foot*

*MSD = Mean Square Distance*
**1.** **begin****2.**    **if** Find *Space_f_*
**then****3.**     **switch** Angle Calculation Method **do****4.**        **case** Center of Gravity**5.**         **if** Find *Gravity, AND Gravity_h_*
**then****6.**              Calculate angle between *Gravity_b_* and *Gravity_h_* based on *Space_f_***7.**         **else****8.**              **return** Get Data**9.**        **case** Total Least Square**10.**          **if** Find a linear function that minimizes *MSD*
**then****11.**            Calculate linear function angle based on *Space _f_***12.**          **else****13.**            **return** Get Data**14.**       Calculate the angle between *Base line* and *calculated angle***15.**       Generalize foot pressure angle using *Rotation matrix***16.**  **else****17.**       **return** Get Data**18.**  **return** success to generation

The second preprocessing step breaks down as follows: we analyze the rotated foot pressure to determine how many footfalls are detected by the footpad, then isolate each footfall, and finally distinguish whether the detached foot pressure is left or right. The reason for isolating the foot pressure to separate left and right footfalls is that the user is recognized by a single machine learning model, and distinguishing between the user’s left and right foot requires much more data and a more complex model than a machine learning model for user recognition only. Therefore, after using a simple algorithm to distinguish the left and right foot, separate user recognition models for foot pressure on the left foot and the right foot can be used to recognize the user efficiently while using fewer data and simpler models.

Algorithm 2 shows the isolation of individual foot pressures and subsequent classification as either left or right foot, expressed in pseudocode. The isolation procedure begins with finding the center line of the foot pressure. This attribute can be used to separate footfalls by selecting any line of space between the first and second footfall, not simply the center between the two foot-pressure end boundaries. The method for judging a footfall to have occurred through separated foot pressure is to locate the arch between the toe and sole of the foot. Once a footfall is determined to have occurred, the foot is distinguished by using the fact that the arch is convex to the left for the left foot and convex to the right for the right foot.

After completion of the preprocessing process shown in Algorithms 1 and 2, the system extracts key features of the foot pressure, which vary from person to person. Using the extracted features, machine learning for user recognition is performed by selecting one of the following as a user-recognition algorithm: fuzzy, GNB, K-NN, or ANN. Thus, in this study we are comparing performance on the Raspberry Pi 3, which is a small embedded system, for two algorithms for measuring the user’s foot angle and four algorithms for recognizing the user.

**Algorithm 2.** Pseudo code for separation and classification of foot pressure.
*Center_i_= Center line of foot pressure*

*Space_f_ = Space between toes and ball of foot*
**1.** **begin**2.    **if** Find *Center_i_*
**then**3.     **if** Find *Space_f_* on left side **then****4.**        **if** Is arch of foot convex to the left? **then**5.         Foot on left side is left foot6.        **else**7.         Foot on left side is right foot8.     **else if** Find *Space_f_* on right side **then**9.        **if** Is arch of foot convex to the right? **then**10.          Foot on right side is right foot**11.**        **else**12.          Foot on right side is left foot13.      **else**14.        **return** Get Data**15.**    **return** Success to classification16. **end**

## 4. Preprocessing and Feature Extraction

For this paper, the proposed preprocessing and feature extraction procedures are as follows. First, calculate the difference in angle for the pressure distribution, based on the orientation of the Raspberry Pi 3 and its joint. Second, rotate the foot pressure by a different angle for generalization. Third, separate the specific footfall from the generalized foot pressure, and classify as either left or right foot. Fourth, extract key foot-pressure features.

### 4.1. Foot-Pressure Angle Measurement

For this study, the measurement method for foot-pressure angle comprises a center-of-gravity determination and a smallest mean square distance calculation, as described above. Section 4.1.1 describes the method for angle measurement using a center of gravity algorithm, while Section 4.1.2 describes the total least square calculation.

#### 4.1.1. Measuring Foot-Pressure Angle Using the Center of Gravity

If the foot-pressure angle is measured only by center of gravity, an error occurs based on the reference point. Therefore, we locate the arch between the toe and sole of the foot and measure how the current foot pressure is directed on the footpad.

##### Locating the Arch of the Foot

**Figure 3:**
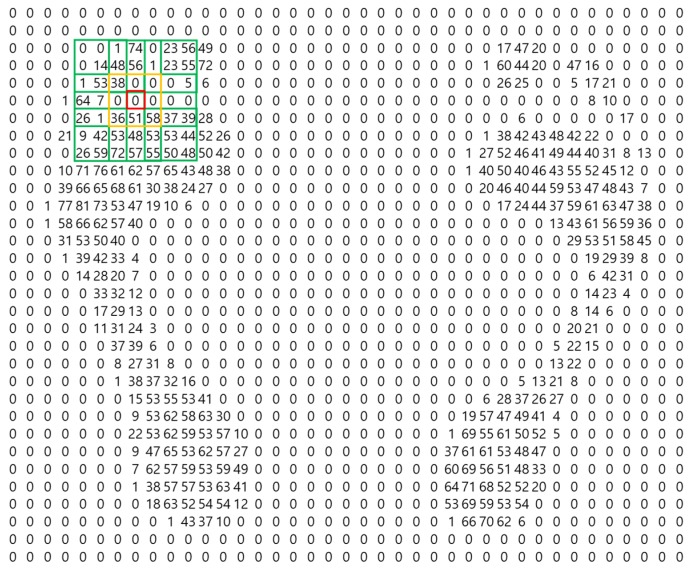
Candidate search for the arch between toe and sole of foot candidate search.

The method for locating the arch of the foot is as follows. First, determine whether the value at the current position is 0, and whether there is a nonzero value in the vicinity. Second, to determine whether or not the current position corresponds to the arch, locate a point two spaces away from the present position. Then, determine if there is a non-zero value in the vicinity that includes the point and place it in the candidate group for the arch of the foot. Figure 3 illustrates this.

**Figure 4:**
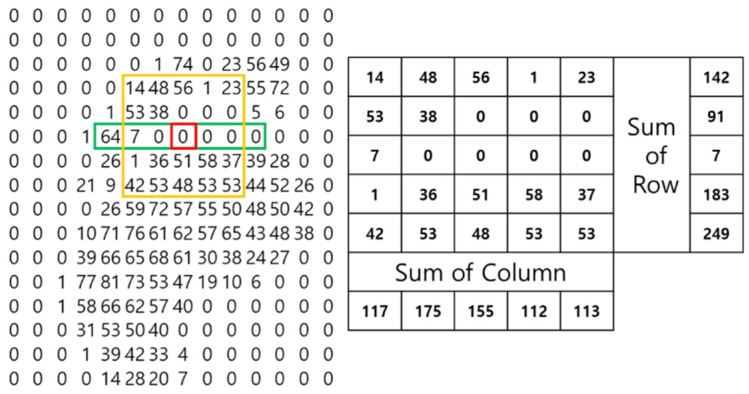
Characteristic pressure features of the arch.

The red box in Figure 3 is the current position; it is determined whether the sum in the vicinity (yellow box) is nonzero based on this position. Also to be determined is whether the sum of all points within two sensors of the current position and the surrounding area (all the green boxes) is zero or not. If the sums of the yellow box and of the green boxes are both nonzero, then this position is a candidate for the arch. The final selection of candidates is made using specific pressure features of the arch. The arch of a foot is characterized by a strong pressure at the toe, then a gradual decrease to the arch itself, and finally a gradual increase in pressure from the arch to the sole of the foot. After applying these features to a candidate group, a judgment is made as to whether the candidate group is the arch or not. This is illustrated in Figure 4.

The red box in Figure 4 creates a 5 by 5 matrix of points (the yellow box in Figure 4) within a distance of two sensors of the current position of the red box and determines whether it matches the following pattern:

1. The sum of the first row (142) is larger than the sum of the second row (91).
2. The sum of the second row (91) is larger than the sum of the third row (7).
3. The sum of the fourth row (183) is larger than the sum of the third row (7).
4. The sum of the fifth row (249) is larger than the sum of the fourth row (183).

If the current position matches this pattern, it is judged to locate the arch. Also, if the column sums rather than the row sums match this pattern, this position is also determined to be the location of the arch.

##### Locating Centers of Gravity

**Figure 5:**
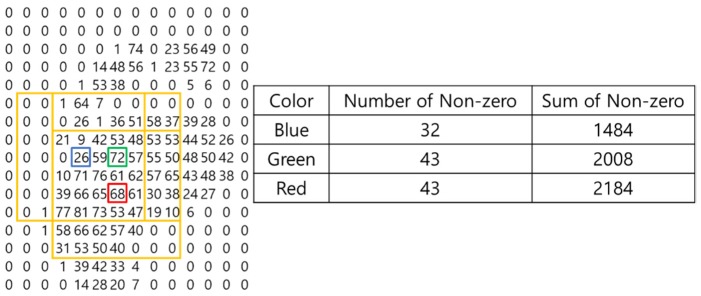
Locating a candidate center of gravity.

The foot-pressure angle is measured as the angle between the center of gravity of the front part of the foot and the center of gravity of the rear part. Therefore, locating centers of gravity is critical to the whole process. This paper uses a heap data structure [26] based on a complete binary tree to find centers of gravity. The primary key value of the heap data structure is the number of nonzero values in the 7 by 7 matrix based on the current position. If the number of nonzero values is the same, set the sum of all values of the 7 by 7 matrix to the secondary key and input it to heap. The parent and child nodes of the heap data structure are set to the centers of gravity at the sole and heel of the foot. This is shown in Figure 5.

**Figure 6:**
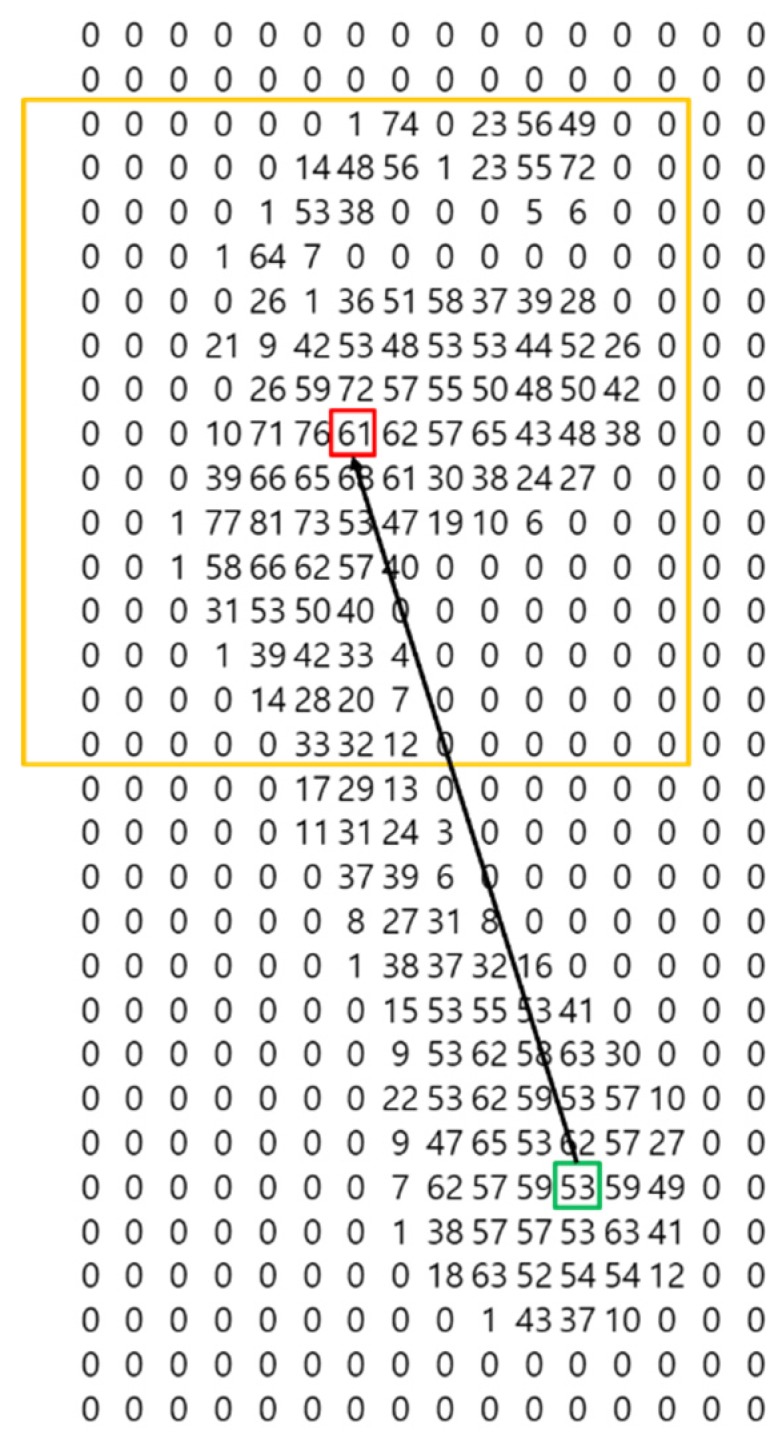
Angular measurement based on centers of gravity.

With the blue box in Figure 5 showing the first position, there are 32 nonzero values in the vicinity (yellow box centered on blue), while there are 43 nonzero values in the vicinity of the green box indicating the second position (yellow box centered on green). The green box is selected as the parent node because there are many nonzero numbers in its vicinity, while the blue box is selected as the child node. However, the number of nonzero values around the red (third position) and green boxes is the same, 43. In such a situation, the parent node is set based on having the larger sum of 43. Therefore, the position of the red box becomes the parent node. Continuing in this way, the parent node becomes the center of gravity at the front of the foot; the child node is set at the center of gravity at the heel of the foot. The angle between the parent node and the nearest child node is measured between the heap data structures thus created. Because the measured angle can change by 180° depending on the reference point, the angle is measured based on the position where the distance between the measured toe point and the center of gravity is large. This is shown in Figure 6.

#### 4.1.2. Total Least Squares

**Figure 7:**
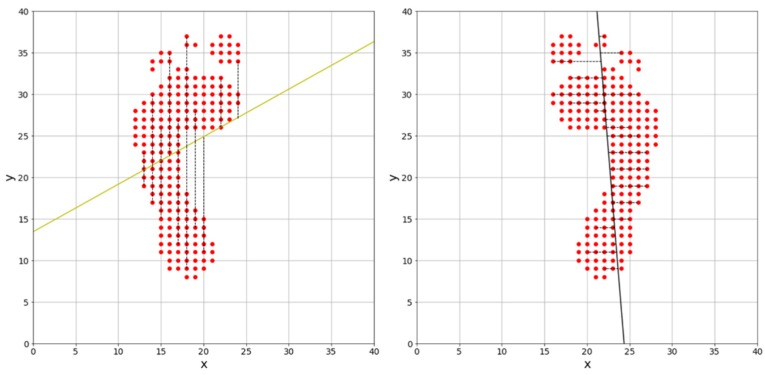
Comparison of least square calculations referenced to *x*- or *y*-axis.

Least squares (LS) [27] determines the linear function with the smallest mean square distance between the measured two-dimensional foot pressure and that linear function. However, LS has different linear functions associated with it because the error is measured in different ways depending on whether the reference line is the *x*-axis or the *y*-axis, as shown in Figure 7.

Figure 7 shows that the same data can have different linear functions associated with it, depending on which axis is taken as the reference line. In this study, the method of deriving the principal components (PCs) using principal component analysis (PCA) [28,29] is applied. PCs are saved in PCA in total least square (TLS) or orthogonal least square (OLS) format, in order to find an *N*-dimensional vector with minimal mean square distance between the original dataset and this *N*-dimensional vector as projected onto the original dataset. This is equivalent to finding the *N*-dimensional vector with the largest variance out of the data projected onto it [30].

**Figure 8:**
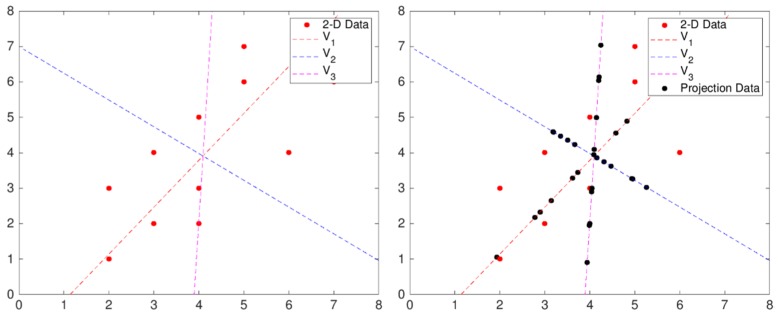
Scatter diagram and result of data projection on principal component vector.

For example, Figure 8 shows three one-dimensional vectors—two principal component vectors and the result as projected on each vector—in a two-dimensional data scatter plot. The projected result shows that the variance of the principal component vector **v**_1_ with the smallest mean square distance from the original dataset is the largest, the variance of the second principal component vector **v**_2_ orthogonal to the principal component vector **v**_1_ is second largest, and the arbitrary vector **v**_3_ has the smallest variance. Thus, vector **v**_1_, which has the largest variance, becomes the principal component vector for the two-dimensional data in Figure 8. We obtain principal component vectors as follows:

(1) $$\begin{array}{c} DataX\in R^{n\times d}\\ \mathrm{Unit}\;\mathrm{Vector}\;=\;\vec{e}\left(UnitVectorshapeisd\times a\right)\\ CovarianceX=\sum \\ IftheXisorthogonalprojection\vec{e},\\ X\vec{e}\in R^{n\times a} \end{array}$$

The data matrix is represented by $X\in R^{n\times d}$ (n is the number of data points) and an arbitrary unit vector which determines orthogonal projection, represented by the vector $\vec{e}$ of dimension d×a, where d-dimensional data is reduced to dimension a ((d>a)) through orthogonal projection. This can be expressed by Equation (1). Therefore, the variance of the $\vec{e}$ vector is obtained by Equation (2).

(2) $$Var\left(X\vec{e}\right)=\frac{1}{n}\overset{n}{\underset{i=1}{\sum }}{\left(X\vec{e}-E\left(X\vec{e}\right)\right)}^{2}$$

Assuming that the average of each column of the *X* matrix $E\left(X\vec{e}\right)$ is 0, the result in Equation (3) can be obtained. In addition, if Equation (3) is developed as Equation (4), we obtain the result in Equation (5).

(3) $$\begin{array}{l} \;Ifeachrowaverageis0,\\ Var\left(X\vec{e}\right)=\frac{1}{n}\overset{n}{\underset{i=1}{\sum }}{\left(X\vec{e}\right)}^{2}=\frac{1}{n}{\left(X\vec{e}\right)}^{T}\times X\vec{e} \end{array}$$

(4) $$\begin{array}{l} Var\left(X\vec{e}\right)=\frac{1}{n}{\left(X\vec{e}\right)}^{T}\times X\vec{e}=\frac{1}{n}{\vec{e}}^{T}X^{T}X\vec{e}\\ \;\frac{1}{n}{\vec{e}}^{T}\left(X^{T}X\right)\vec{e}={\vec{e}}^{T}\left(\frac{X^{T}X}{n}\right)\vec{e\;}=\;\sum \vec{e} \end{array}$$

(5) $$\underset{e}{max}\left\{Var\left(X\vec{e}\right)\right\}=\underset{e}{max}{\vec{e}}^{T}\sum \vec{e}$$

There are numerous possibilities for a vector $\vec{e}$ satisfying Equation (5). Since the variance increases simply by increasing the length of the $\vec{e}$ vector, the Lagrange multiplier method [31] can be used to construct the constraint of Equation (6), which can then be used to derive the Lagrange function Equation (7). To obtain the maximum value, we can derive Equation (8) by differentiating L with respect to $\vec{e}$.

(6) $$\|\vec{e}\|={\vec{e}}^{T}\vec{e}=1$$

(7) $$L={\vec{e}}^{T}\sum \vec{e}-\lambda \left({\vec{e}}^{T}\vec{e}-1\right)$$

(8) $$\frac{\partial L}{\partial \vec{e}}\left({\vec{e}}^{T}\sum \vec{e}-\lambda \left({\vec{e}}^{T}\vec{e}-1\right)\right)=2\sum \vec{e}-2\lambda \vec{e}=\vec{e}\left(\sum -\lambda \right)=0$$

In Equation (8), $\vec{e}$ is the eigenvector of the covariance matrix ∑ and λ is an eigenvalue of ∑ according to the definition of an eigenvector.

**Figure 9:**
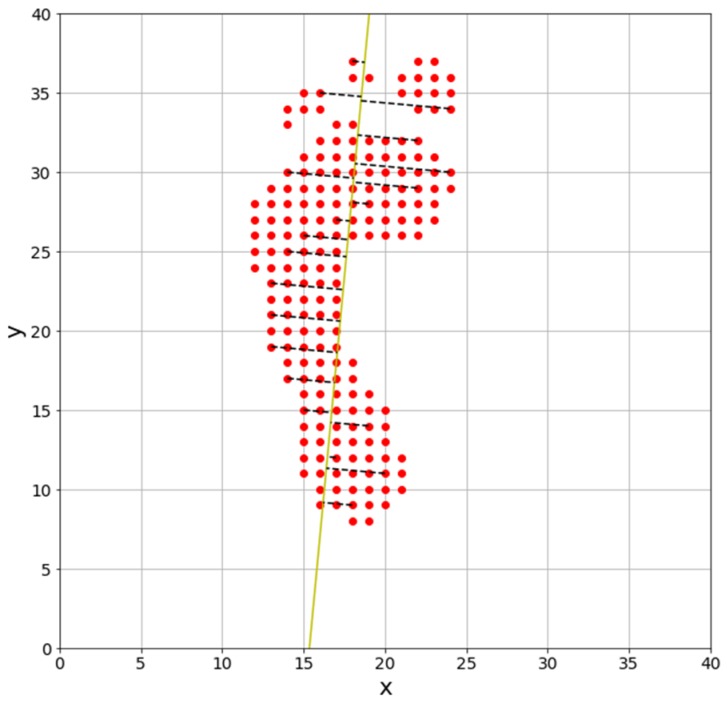
Angular measurement result using total least squares procedure.

In this study, the principal component vector is derived by locating the center of gravity of foot pressure at (0, 0), finding the covariance matrix of the foot pressure’s data matrix, and then finding the eigenvector of the covariance matrix with large eigenvalue. The result is shown in Figure 9.

Figure 9 shows the angle at which the error is minimized, but there could be an error of 180° due to the choice of reference point. As previously mentioned, we overcome this error by measuring the angle in the clockwise direction, based on the arch of the foot.

### 4.2. Rotation of Foot Pressure

**Figure 10:**
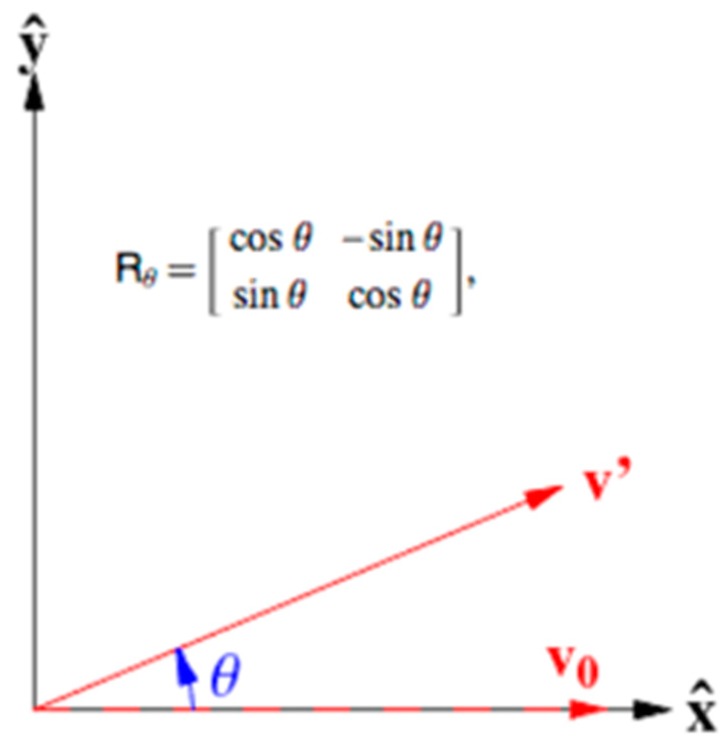
Rotation of data using a rotation matrix.

After measuring the angle, we use a rotation matrix to generalize the foot pressure. The rotation matrix rotates a two-dimensional foot-pressure data matrix about a central reference point. In this paper, generalization of the foot pressure is performed by setting the reference point as the center of the footpad, at (24, 24), as shown in Figure 10.

### 4.3. Separation and Classification of Foot Pressure

It is not easy to ensure high accuracy using machine learning algorithms with low data and low computing power. In order to increase accuracy under these conditions, the rotated footfalls are separated during the preprocessing process and each separated part is classified as either the left or the right foot. The separation method consists in finding the start and end points of the foot for left and right, and then using the average of the points where the first foot ends and where the second foot begins, respectively. We locate the starting point of a foot as follows.

1. The current position has a value of zero.
2. The sum of the values at the eight cardinal points of the current position is nonzero.
3. The sum for the eight cardinal points of the previous position is zero.

The rearmost point of a foot is located as follows.

1. The current position has a value of zero.
2. The sum of the values at the eight cardinal points of the current position is zero.
3. The sum for the eight cardinal points of the previous position is nonzero.

**Figure 11:**
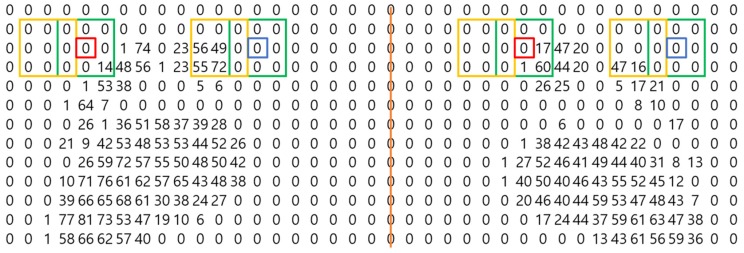
Locating the start and end points of the foot.

The red box in Figure 11 is determined as the starting point of the foot if the sum of values at the eight cardinal points of the current position (the green box) is not zero, and the sum of the eight cardinal points of the previous position (the yellow box) to the left of the current position is zero. Also, if the current position (blue box) has a zero value, the sum for the eight cardinal points of the current position (green box) is 0, and the sum for the eight cardinal points of the previous position (yellow box) is nonzero, then the current position is judged to be the foot’s rearmost point. Therefore, we divide the foot pressure data by using the average (orange line) of red and blue boxes in Figure 11.

After separating foot-pressure data, foot classification takes advantage of the characteristics of the arch of the foot. The arch is characterized by a left-handed convexity in the left foot and a right-handed convexity in the right foot. Thus, the left side of the left foot is flat, so deviation is small, while the right side is concave, so deviation will be large. Based on these characteristics, the foot classification procedure in this paper is as follows:

1. Calculate the deviation on the left of the widest part of the front of the foot and on the left of the narrowest part of the center of the foot.
2. Calculate the deviation from the left of the widest part of the heel to the left of the narrowest part of the center of the foot.
3. Calculate the deviation on the right side in the same way.
4. If the deviation on the left side is larger than the deviation on the right side, classify this as a right foot. If the deviation is larger on the right side is larger, classify as a left foot.

**Figure 12:**
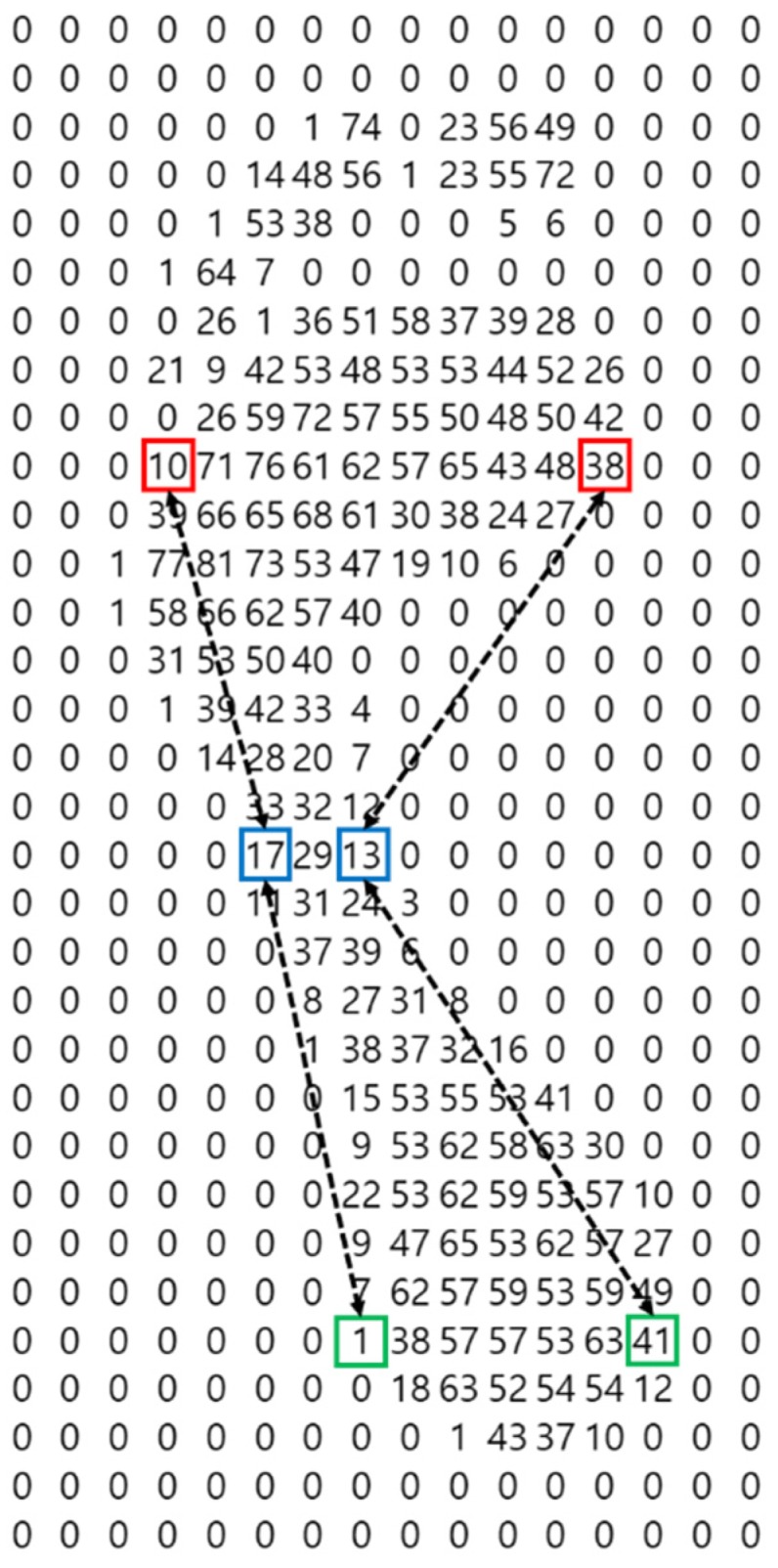
Deviation between left and right sides of a footfall.

This procedure is illustrated in Figure 12.

The red boxes in Figure 12 correspond to the left and right sides of the widest part of the front of the foot, the blue boxes to the left and right sides of the narrowest part of the center of the foot, and the green boxes to the left and right sides of the widest part of the heel. The difference between the red box and the blue box on the left of Figure 12, plus the difference between the green box and the blue box, is 4, while the deviation on the right is 9. Therefore, this foot is distinguished as a left foot because the deviation on the right side is larger.

### 4.4. Feature Extraction

In this paper, we define nine features for each of the left and right foot.

1. The width of the foot
2. The length of the foot
3. The width of the widest part of the front of the foot
4. The length from the widest point ③ of the front of the foot front to the front end ① of the foot
5. The minimum width at the center of the foot
6. The length from the narrowest point ⑤ of the center of the foot to the widest point ③ of the front of the foot
7. The maximum width of the heel
8. The length from the widest point ⑦ of the heel to the narrowest point ⑤ of the center of the foot
9. The length from the widest point ⑦ of the heel to the widest point ③ of the front of the foot

**Figure 13:**
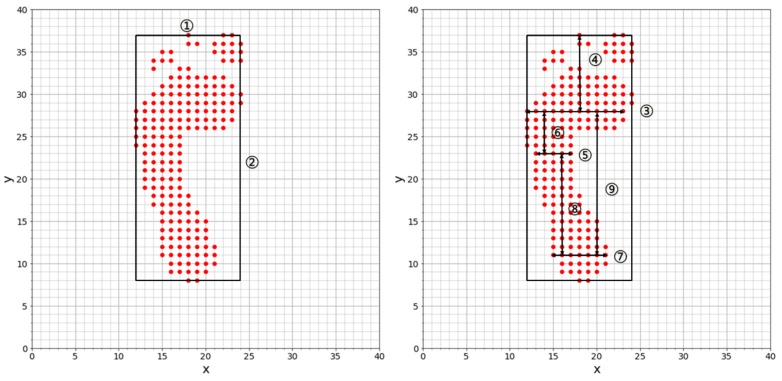
Nine features derived from foot pressure.

Figure 13 shows these nine features.

## 5. Performance Comparison of User Recognition Algorithms

In this study, the foot pressure dataset with six data samples selected to be used in the experiment consists of a data set preprocessed by center of gravity rotation and a data set preprocessed with TLS. Each data set contains 80 data points per person generated by preprocessing foot-pressure data measured at the four cardinal points (20 times per direction). Our model classifies the left and right feet in the preprocessing stage, separating the user recognition models for left and right feet. To measure the performance of the model, the model performance for each foot was evaluated as the average of five sets of cross-validation results, and the performance of the user recognition model was then evaluated as the average of the model performances for each foot. The ratio of the learner data set to the execution data set was set to 8:2, and the ratio of the data set per person to the data set of the four cardinal points was set to be equal.

### 5.1. Fuzzy Theory

**Figure 14:**
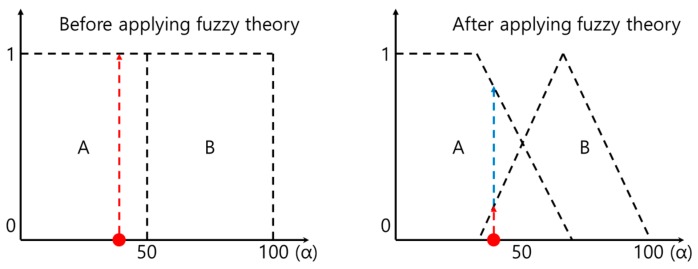
The many-valued logic of fuzzy theory.

Fuzzy theory began with the concept of accepting ambiguous middle values instead of the dichotomous 1-0 logic characteristic of existing computers, expressing such values via many-valued logic. So, for example, a characteristic value of α is expressed using weights as A 70, B 30, not as either A or B. Figure 14 illustrates this concept.

In this study, weights are assigned differently for each feature, based on standard deviation. The standard deviation indicates how far the data are scattered from the average, so if the standard deviation is small, the data are clustered tightly. Here, we calculated averages of the features for each user and then calculated the standard deviations for these averages to determine how much the features were scattered. Features with large standard deviations gave high weights and those with small standard deviations gave low weights. We also set the range of the fuzzy graph to be (standard deviation) × 10 to cover the full possible range of feature values.

**Figure 15:**
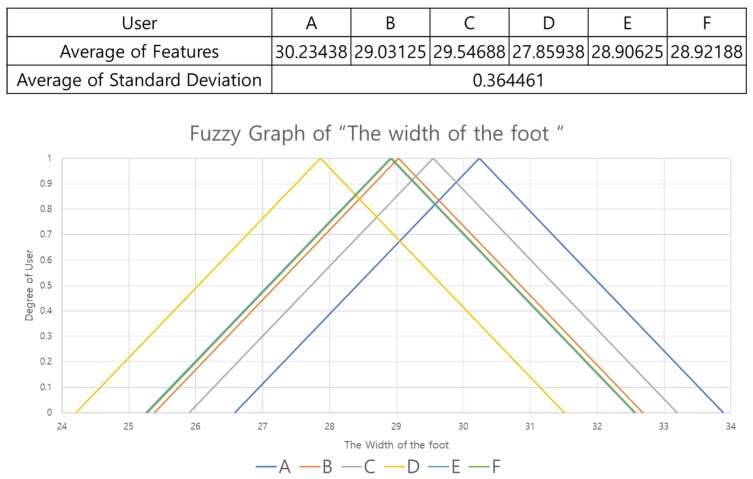
Fuzzy graph of the ‘width of foot’ feature.

Figure 15 is a fuzzy graph of the ‘width of foot’ feature. In a fuzzy graph that expresses one feature, it is difficult to distinguish, for instance, user D from user E because the averages are very similar. For this reason, we use nine features and set different weights for each feature to model significance so as to output users with highest probability.

**Figure 16:**
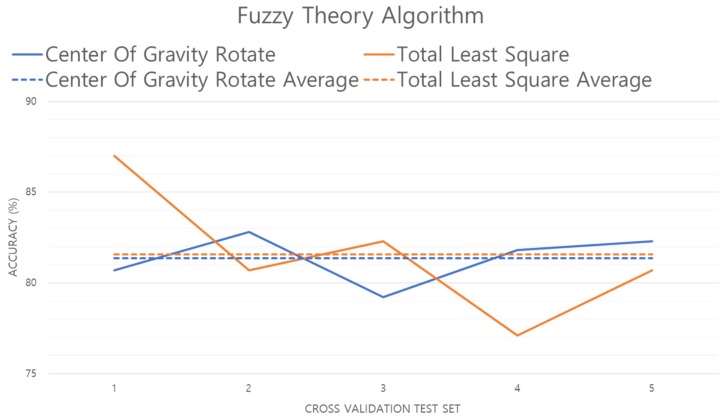
Performance evaluation of the fuzzy algorithm.

Figure 16 shows the performance evaluation of the fuzzy algorithm. As shown in the results, the accuracy of the TLS method is improved by 0.2% compared to the center of gravity rotation method. This is because the TLS method performs better generalization of the foot pressure than the center of gravity method, and extracts features with low standard deviation.

### 5.2. Gaussian Naive Bayes (GNB) Method

**Figure 17:**
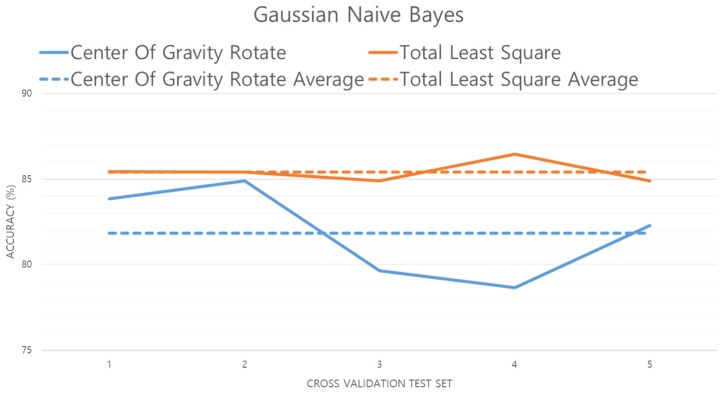
Performance evaluation of the GNB algorithm.

GNB is a method for applying a Bayesian algorithm given the assumption that the feature collected from the preprocessed data comprises continuous data with Gaussian distribution. GNB is a fast, easy to implement, and relatively high-performance classifier. Therefore, we chose to study it as the best machine learning algorithm for a real-time user recognition system. However, GNB tends to degrade if the basic independence hypothesis is not supported. That is, once data with a large error are input, the performance rapidly deteriorates [15]. In this study, we experimented with GNB with the same prior probability and used the GNB model implemented in Python’s scikit-learn package [32]. The results of the experiment are shown in Figure 17.

Figure 17 shows the performance evaluation of the GNB model. The accuracy of the TLS method has increased by 3.57% over the center of gravity method. This means that data preprocessed by the TLS method has little error data from the average compared to the data preprocessed by the center of gravity method. That is, the data preprocessed by the TLS method is better generalized.

### 5.3. K-Nearest Neighbor (K-NN)

The K-NN classifier finds the *k* closest neighbors of the pressure distribution with the highest similarity and classifies this as the most strongly weighted group. The advantage of the K-NN classifier is that it performs more accurately than the GNB algorithm, but it has the disadvantage of slow processing speed for large learning sets [16]. However, since the proposed user recognition system does not recognize many people, it does not require a large learning data set. Therefore, if the K-NN algorithm is used in this system, the processing speed will not be slow enough to impede real-time recognition. The K-NN algorithm model used in this system is the one implemented in scikit-learn [32].

**Figure 18:**
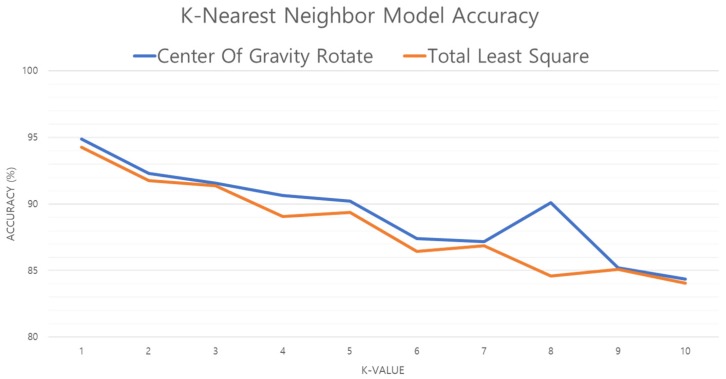
Performance evaluation of K-NN model against *k* (nearest neighbors).

The K-NN algorithm varies in accuracy and processing speed depending on the value of *k*. Therefore, the K-NN model with optimal *k* is the optimal K-NN model. Figure 18 shows the accuracy of the K-NN model as the value of *k* is increased. When *k* is 1, Err (1-NN) ≤ 2 × Ideal Err (error of the ideal model, which is suitable for given data) proves [33,34] that the model performance is guaranteed over 94%, and that increasing the value of *k* results in lower accuracy of both center of gravity and TLS methods. Thus, in this study, performance is compared using a model with *k* set to 1.

**Figure 19:**
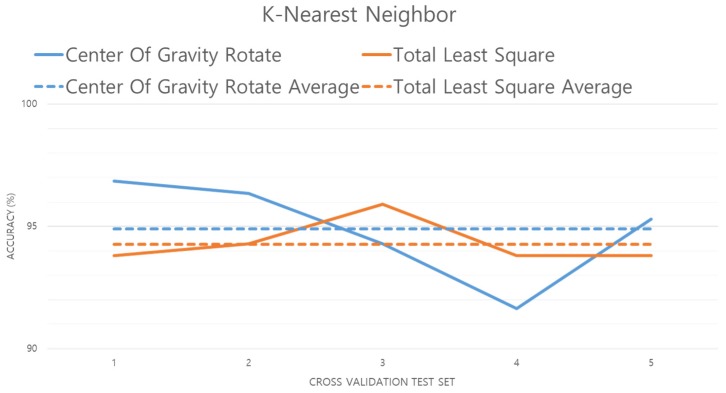
Performance evaluation of K-NN algorithm.

Figure 19 compares the results of the experiment with the data preprocessed by the center of gravity and TLS methods. The results show that TLS is about 0.5% less accurate than other user perception algorithms. This is because the K-NN algorithm is not affected as much by variance as GNB or fuzzy theory, but is influenced by the number (*k*) of nearest distant features. In other words, if the difference between the features is not large, the accuracy is lowered because the number of errors with TLS is small. K-NN is also susceptible to the curse of dimensionality: if dimensional reduction is applied, higher accuracy will result [35,36].

### 5.4. Artificial Neural Network (ANN)

Artificial neural networks are designed to mimic the working principles of the human brain and neurons. An ANN is made up of connections among nodes that mimic neurons and consists of one input layer, one or more hidden layers, and one output layer. The speed depends on the size of the hidden layer(s) and the number of repetitions [37,38]. This paper uses the ANN model implemented in scikit-learn [32] and consists of nine characteristics in the input layer and the user in output layer. The model was constructed by selecting the optimal hidden layer (*M* × *M* × *M*) and Max_Iteration with Learning_Rate = 0.001, Activation = ‘Relu’, Optimizer = ‘Adam’, and Max_Iteration = *N*.

**Figure 20:**
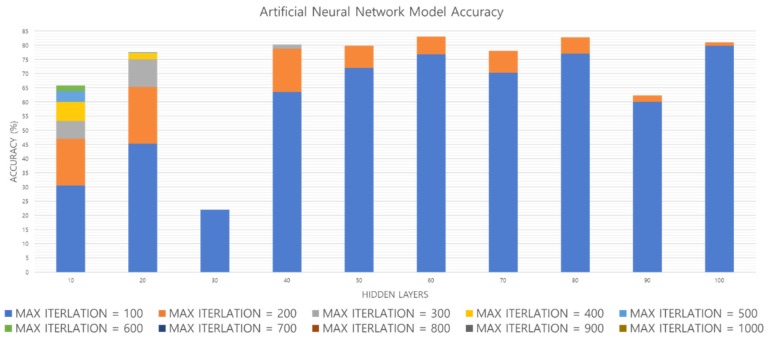
ANN model performance evaluation based on accuracy.

**Figure 21:**
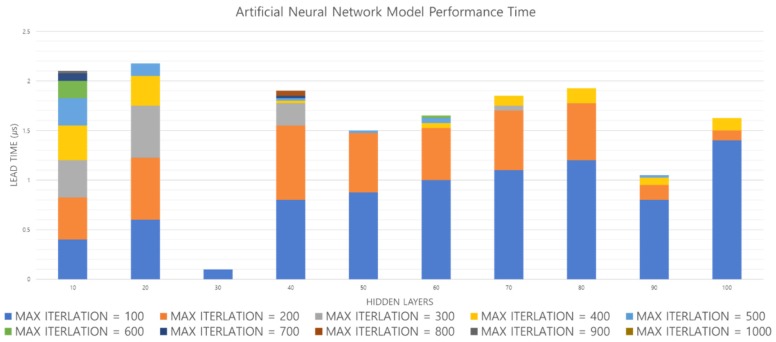
Performance evaluation of ANN model based on performance time.

Figure 20 shows the accuracy obtained by increasing the hidden layer and Max_Iteration of the ANN model. Figure 21 shows the time from the input of one footfall to the output. As shown in Figure 20, when the hidden layer = 10, accuracy increases when Max_Iteration increases. However, with the hidden layer = 50, the highest accuracy is obtained when Max_Iteration = 200, and finally the highest accuracy of the ANN model is obtained when the hidden layer = 80 and Max_Iteration = 200. In Figure 21, when the hidden layer = 80 and Max_Iteration = 200, the performance time is less than 2 µs, so both the accuracy and the performance time are satisfied. Therefore, we compare performance using an ANN model with hidden layer = 80 and Max_Iteration = 200.

**Figure 22:**
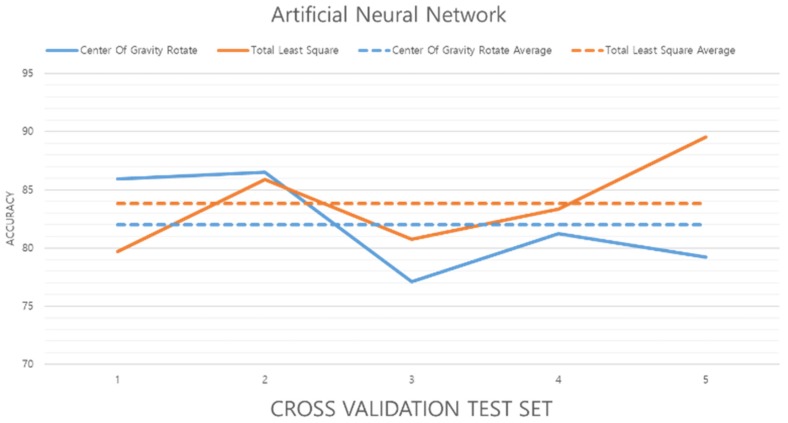
Performance evaluation of the chosen ANN algorithm.

Figure 22 shows the results of the experiment using data preprocessed by the center of gravity and TLS methods. The results show that TLS method accuracy is increased by about 1.8%.

### 5.5. Determinism Evaluation

There are two important points to consider in this study. The first is guaranteed determinism in small embedded systems. That is, in a small embedded system, the calculation must be completed within a certain time, and the time given in the system is from the moment the dementia patient or senior citizen steps on the footpad to the time when the foot lifts off the foot pad. Therefore, since foot-pressure measurement using the footpad is 20 Hz, the maximum performance time must be 50 µs in order to recognize the user before the next foot pressure distribution. If it is longer than 50 µs, the next foot pressure will not allow recognition of the user and it will be necessary to wait until the first subsequent foot pressure after user recognition is complete. Therefore, in order to guarantee determinism, performance time must be guaranteed less than 50 µs.

The second point concerns accuracy. Even if determinism is guaranteed, if the accuracy is too low, the system is useless. Therefore, the highest possible accuracy should be achieved under the condition that determinism is guaranteed. In order to satisfy both conditions, performance evaluation was carried out based on determinism in Raspberry Pi 3, which is a small embedded system. The performance evaluation is as follows:

Table 1 displays a performance evaluation and accuracy on Raspberry Pi 3. The combining center of gravity and KNN accuracy is the highest at 94.89%. Because KNN is an algorithm that compares the similarity by calculating the distance between features. So it is a noise-robust algorithm compared to algorithms. Moreover, in this paper, we set K = 1 to find the nearest neighbors to guarantee the performance of the model. Therefore, KNN accuracy is best of all algorithms. And the combining TLS and ANN is below expectations at 83.85%. Because, for real-time processing in an embedded system environment, we should limit hidden layer size and the number of iterations. Therefore, ANN performance is relatively low. The performance time is the average over ten repetitions of the time taken for one foot pressure, and the maximum performance time is the longest time among these times. Among preprocessing algorithms, the performance time of the center of gravity method is 24.44 µs, while the performance time of the TLS is 25.9 µs. Although the difference is only 1.5 µs, the performance time of TLS depends on the number of data points for foot pressure. Therefore, the maximum performance times differ by 2.2 µs. The user recognition algorithm with the shortest performance time is the fuzzy algorithm, 0.71 µs, while the K-NN algorithm has the longest performance time at 3.87 µs. Among performance times of the whole system combining user recognition algorithm and preprocessing, the system combining center of gravity and fuzzy theory is the fastest at 25.15 µs, while the system with TLS and K-NN is the slowest at 29.77 µs.

**Figure 23:**
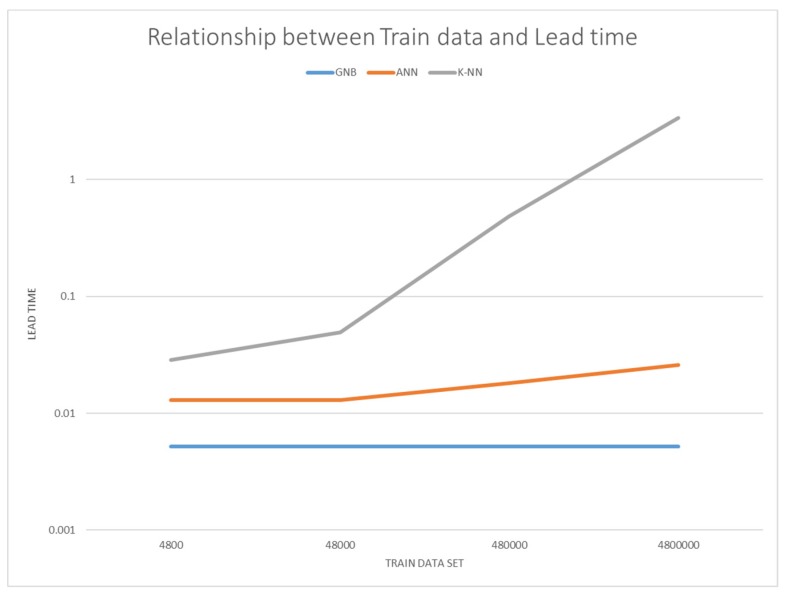
Performance time as a function of amount of learning data.

In this study, the algorithm should be selected to guarantee the highest accuracy under the condition that determinism is guaranteed. If all the algorithms satisfy determinism because they recognize the user within 50 µs, it would be best to use a system with the highest accuracy: one that combines center of gravity rotation and the K-NN algorithm. However, as can be seen in Figure 23, which shows the performance time graph of the user recognition algorithm as the amount of data increases, when the amount of learning data is 4800 the performance time of the K-NN algorithm is 75 µs, but performance time increases rapidly as learning data increases [39,40]. Therefore, proceeding with online learning to learn data in real time is not possible. In this system, we combine TLS and GNB with the second highest accuracy, but with less variation in performance time depending on the amount of data.

## 6. Conclusions

In this study, we propose a system that recognizes the user in real time by measuring the user’s foot pressure on a footpad. This system recognizes users as they simply step onto a footpad, to avoid the disadvantages of existing systems: cumbersome authentication procedures, invasion of privacy, and the necessity of having and using a specific device. For this purpose, the characteristics of the user’s foot pressure are extracted and applied to various user recognition algorithms. We also studied which algorithms guaranteed the greatest determinism by measuring the performance time of various user-recognition algorithms. In our results, a combination of center of gravity and k-nearest neighbor algorithms on a Raspberry Pi 3 computer showed about 95% accuracy for a performance time of about 28 µs.

Future research will recognize patterns of user behavior as well as user recognition by installing footpads in various locations and exploiting communication between units. And we will provide a more reliable service when the foot is partially stepped, in combination with the data analysis technology on the existing smart home platform. Therefore, the security or communication that should be considered important when creating an application will be covered in the future research, not in this research that classification users. As a result, we will be able to identify a user’s health condition and extent of dementia, and furthermore, we will study the behavior patterns of dementia patients to create an environment where they can live independently without constant caregiver supervision.

## Figures and Tables

**Table 1:** Performance evaluation with Raspberry Pi 3.

Accuracy (%) and Performance Time (µs) on Raspberry Pi3								
Preprocessing	Performance Time	Maximum Performance Time	Recognition Algorithm	Performance Time	Maximum Performance Time	Whole System Performance Time	Whole system Maximum Performance Time	Accuracy
Center of gravity	24.44 µs	24.7 µs	FUZZY	0.71 µs	1.2 µs	25.15 µs	25.9 µs	81.36%
			GNB	2.48 µs	2.9 µs	26.92 µs	27.6 µs	81.85%
			K-NN	3.87 µs	5.3 µs	28.31 µs	30 µs	94.89%
			ANN	2.51 µs	3.4 µs	26.95 µs	28.1 µs	82%
TLS	25.9 µs	26.9 µs	FUZZY	0.71 µs	1.2 µs	26.61 µs	28.1 µs	81.56%
			GNB	2.48 µs	2.9 µs	28.38 µs	29.8 µs	85.42%
			K-NN	3.87 µs	5.3 µs	29.77 µs	32.2 µs	94.27%
			ANN	2.51 µs	3.4 µs	28.41 µs	30.3 µs	83.85%
